# 

**Published:** 1889-06-15

**Authors:** 


### A Year's Work in the Hospitals and Medical Charities of London.

The following Table and Figures should be studied with the remarks in the Summary on the last page, to make their bearing more intelligible and better understood.

### NEWINGTON AND SOUTH DISTRICT.

Comprising Battersea, Wandsworth, Tooting, Balham, Streatham, Brixton, Lambeth, Newington, Southwark, Bermondsey, Camberwell, Greenwich, Deptford, Lewisham, Blackheath, Woolwich, etc.

**Special Note.**—It will be seen that out of 1,461 available beds only 948 were daily occupied in this district during 1888. Though the proportion of unoccupied beds is not quite so large as it was last year, one-third of the available Hospital accommodation was not utilized for the relief of the poor for want of funds. As it is, the total income was upwards of £3,000 less than the expenditure entailed by the maintenance of the 948 beds actually occupied throughout the entire year.

| No. of Beds. | No. of Beds Daily Occupied. | Hospitals. | In-patients. | Out-patients. | Total Expenditure. | Income. | | | Total Income. | Legacies not included. |
|---|---|---|---|---|---|---|---|---|---|---|
| | | | | | | Charitable. | Proprietary. | Patients' Payments. | | |
| | | | | | £ | £ | £ | £ | £ | £ |
| 500 | 418 | Guy's ... ... ... ... ... ... | 5,385 | 40,014 | 34,198 | 2,159 | 28,656 | 2,787 | 33,602 | ... |
| 24 | 20 | Miller... ... ... ... ... ... | 226 | 13,787 | 3,439 | 2,362 | 315 | ... | 2,677 | ... |
| 225 | 191 | Seamen's ... ... ... ... | 2,463 | 5,914 | 11,495 | 7,653 | 4,011 | 112 | 11,776 | 1,170 |
| 60 | 46 | Evelina, for Children ... ... | 524 | 5,496 | 6,001 | 3,856 | 692 | 58 | 4,606 | 1,200 |
| 45 | 42 | Home for Sick Children ... ... | 249 | 1,506 | 1,451 | 1,200 | ... | 309 | 1,509 | ... |
| 24 | 19 | General Lying-in ... ... ... | 497 | 911 | 3,259 | 764 | 2,303 | ... | 3,067 | ... |
| 51 | 48 | Royal, for Children and Women... | 450 | 7,633 | 3,889 | 2,957 | 725 | 187 | 3,869 | 250 |
| 8 | 7 | Gordon, for Fistula... ... ... | 130 | 492 | 977 | 462 | ... | 470 | 932 | ... |
| 14 | 10 | Royal South London Ophthalmic.. | 113 | 6,292 | 1,215 | 829 | 118 | ... | 947 | ... |
| 10 | 2 | Hospital for Diseases of the Skin... | 18 | 6,056 | 981 | 601 | 100 | 622 | 1,323 | 380 |
| 500 | 145 | Metropolitan Convalescent .. | 3,068 | ... | 7,024 | 5,535 | 381 | 49 | 5,965 | 1,420 |
| 1,461 | 948 | | 13,123 | 88,101 | 73,929 | 28,378 | 37,301 | 4,594 | 70,273 | 4,420 |
| | | Dispensaries. | | | | | | | | |
| ... | ... | Battersea Provident ... ... | ... | 8,523 | 1,898 | 84 | 30 | 1,831 | 1,945 | ... |
| ... | ... | Brixton, etc... ... ... ... | ... | 4,336 | 623 | 513 | 63 | 80 | 656 | ... |
| ... | ... | Camberwell Provident ... ... | .. | 11,946 | 1,846 | 500 | 75 | 1,311 | 1,886 | ... |
| ... | ... | Clapham ... ... ... ... ... | ... | 1,731 | 423 | 394 | ... | 94 | 488 | ... |
| ... | ... | Forest Hill ... ... ... .. | ... | 1,107 | 560 | 223 | ... | 338 | 561 | ... |
| ... | ... | Gipsy Hill ... .. ... ... | ... | 2,251 | 275 | 60 | ... | 200 | 260 | ... |
| ... | ... | Royal South London ... ... | ... | 6,023 | 714 | 685 | 101 | .. | 786 | 250 |
| ... | ... | South Lambeth, etc. ... ... | ... | 2,583 | 634 | 379 | 6 | 219 | 604 | ... |
| ... | ... | South London Medical Aid ... | ... | 1,123 | 470 | 64 | 94 | 240 | 398 | ... |
| ... | ... | Wandsworth Common ... .. | ... | 624 | 154 | 58 | ... | 95 | 153 | ... |
| ... | ... | West Dulwich ... ... .. | ... | 112 | 41 | 17 | ... | 29 | 46 | ... |
| 1,461 | 948 | | 13,123 | 128,430 | 81,567 | 31,355 | 37,670 | 9,031 | 78,056 | 4,670 |

### CITY AND EAST CENTRAL DISTRICT.

Comprising the City, St. Luke's, Shoreditch, Finsbury, and Clerkenwell.

**Special Note.**—Out of the 663 beds provided by the Hospitals of this district 413 were constantly occupied during 1888, leaving 250 beds empty for want of funds. The actual expenditure amounted to £39,774, and the actual income to only £33,663, leaving a deficiency of £6,111 as a debt upon the Hospitals for the work for the poor of the district during the year 1888, despite the fact that nearly half the available beds were not occupied throughout the whole of the year. This is a terrible record for the wealthiest portion of the wealthiest city in the world.

| No. of Beds. | No. of Beds Daily Occupied. | Hospitals. | In-patients. | Out-patients. | Total Expenditure. | Income. | | | Total Income. | Legacies over £100 not included. |
|---|---|---|---|---|---|---|---|---|---|---|
| | | | | | | Charitable. | Proprietary. | Patients' Payments. | | |
| | | | | | £ | £ | £ | £ | £ | £ |
| 160 | 25 | Metropolitan... ... ... ... | 255 | 11,500 | 4,506 | 3,202 | 128 | 568 | 3,898 | 700 |
| 160 | 133 | Royal Free ... ... ... ... | 1,823 | 21,676 | 10,853 | 7,212 | 1,078 | ... | 8,290 | 5,038 |
| 80 | 46 | Royal Hos'l for Diseases of the Chest | 418 | 5,465 | 4,820 | 2,836 | 357 | ... | 3,193 | 1,700 |
| 55 | 47 | North-Eastern, for Children ... | 728 | 13,754 | 4,476 | 4,108 | 235 | 694 | 5,037 | ... |
| 32 | 18 | City of London Lying-In ... ... | 410 | 1,354 | 3,454 | 646 | 2,740 | 25 | 3,411 | ... |
| 34 | 20 | St. Mark's, for Fistula ... ... | 297 | 905 | 2,246 | 1,364 | 939 | ... | 2,303 | 1,508 |
| 100 | 95 | Royal London Ophthalmic ... | 2,196 | 25,261 | 6,157 | 2,819 | 1,177 | ... | 3,996 | 1,356 |
| 25 | 16 | City Orthopædic ... ... ... | 130 | 2,425 | 1,386 | 1,592 | 66 | ... | 1,658 | ... |
| 17 | 14 | Central London Throat and Ear... | 340 | 5,845 | 1,876 | 660 | 32 | 1,185 | 1,877 | ... |
| 663 | 413 | | 6,597 | 88,185 | 39,774 | 24,439 | 6,752 | 2,472 | 33,663 | 10,302 |
| | | Dispensaries. | | | | | | | | |
| ... | ... | City ... ... ... ... ... ... | ... | 4,693 | 1,261 | 932 | 149 | ... | 1,081 | 180 |
| ... | ... | City of London and East London | ... | 7,429 | 792 | 117 | 23 | 749 | 889 | ... |
| ... | ... | Farringdon General ... ... | ... | 7,536 | 634 | 470 | ... | 344 | 814 | ... |
| ... | ... | Finsbury ... ... ... ... ... | ... | 17,751 | 819 | 674 | 106 | 289 | 1,069 | 200 |
| ... | ... | Metropolitan ... ... ... | .. | 6,396 | 619 | 399 | 168 | 219 | 786 | ... |
| ... | ... | Royal General ... ... ... | ... | 3,970 | 862 | 455 | 246 | 102 | 803 | ... |
| ... | ... | Royal Maternity Charity ... ... | ... | 3,604 | 2,551 | 774 | 1,087 | ... | 1,861 | ... |
| 663 | 413 | | 6,597 | 139,564 | 47,312 | 28,260 | 8,531 | 4,175 | 40,966 | 10,682 |

### WESTMINSTER DISTRICT.

Comprising Westminster City and Liberties.

**Special Note.**—The Hospitals in this district contain 863 beds, of which 702 were constantly occupied throughout 1888, leaving 164 unused for want of funds. The deficiency which the managers have to face this year is £10,646, which is very heavy.

| No. of Beds. | No. of Beds Daily Occupied. | Hospitals. | In-patients. | Out-patients | Total Expenditure. | Income. | | | Total Income. | Legacies over £100 not included. |
|---|---|---|---|---|---|---|---|---|---|---|
| | | | | | | Charitable. | Proprietary. | Patients' Payments. | | |
| | | | | | £ | £ | £ | £ | £ | £ |
| 175 | 141 | Charing Cross ... ... ... | 1,870 | 19,352 | 14,439 | 7,518 | 3,436 | ... | 10,954 | ... |
| 206 | 162 | King's College ... ... ... | 1,955 | 18,395 | 17,598 | 10,965 | 1,756 | 8 | 12,729 | 2,080 |
| 205 | 176 | Westminster... ... ... ... | 2,684 | 22,359 | 12,323 | 5,435 | 2,722 | ... | 8,157 | 4,380 |
| 132 | 114 | Ventnor, for Consumption ... | 735 | ... | 9,280 | 4,490 | 1,530 | 2,894 | 8,914 | ... |
| 14 | 10 | Grosvenor, for Women & Children | 69 | 2,295 | 1,140 | 681 | 13 | 442 | 1,136 | ... |
| 50 | 24 | Royal Westminster Ophthalmic... | 324 | 7,571 | 1,796 | 1,718 | 365 | 61 | 2,144 | 720 |
| 50 | 50 | Royal Orthopædic ... ... ... | 155 | 1,112 | 2,283 | 2,558 | 409 | 113 | 3,080 | 1,150 |
| 16 | 10 | St. John's, for Disease of the Skin | 122 | 4,974 | 2,852 | 1,622 | 47 | 1,142 | 2,811 | ... |
| 18 | 15 | Hospital for Diseases of the Throat | 314 | 6,516 | 2,503 | 831 | 51 | 2,192 | 3,074 | 450 |
| ... | ... | Dental ... ... ... ... ... | ... | 31,682 | 1,800 | 1,948 | 175 | 246 | 2,369 | 2,000 |
| 866 | 702 | | 8,228 | 114,256 | 66,014 | 37,766 | 10,504 | 7,098 | 55,368 | 10,780 |
| | | Dispensaries. | | | | | | | | |
| ... | ... | Public... ... ... ... ... | ... | 3,604 | 840 | 420 | 202 | ... | 622 | 150 |
| ... | ... | St. George and St. James... ... | ... | 3,394 | 743 | 528 | 5 | 34 | 567 | 1,000 |
| ... | ... | St. George, Hanover Square ... | ... | 1,811 | 643 | 565 | 20 | 134 | 719 | ... |
| ... | ... | Western ... ... ... ... | ... | 3,437 | 920 | 337 | 415 | 254 | 1,006 | 1,003 |
| ... | ... | Westminster General ... ... | ... | 4,857 | 729 | 383 | 146 | 52 | 581 | .. |
| 866 | 702 | | 8,228 | 131,359 | 69,889 | 39,999 | 11,292 | 7,572 | 58,863 | 12,933 |

### ST. MARYLEBONE AND WEST CENTRAL DISTRICT.

Comprising St. Marylebone, St. John's Wood, Bloomsbury, Holborn, etc.

**Special Note.**—Of the 1,225 beds this district contains, 937 only were in daily use during the year 1888. The total expenditure amounted to £77,995, and the income to only £70,617, leaving a deficiency of over £7,000 to be borne by the Hospitals.

| No. of Beds. | No. of Beds Daily Occupied. | Hospitals. | In-patients. | Out-patients. | Total Expenditure. | Income. | | | Total Income. | Legacies over £100 not included. |
|---|---|---|---|---|---|---|---|---|---|---|
| | | | | | | Charitable. | Proprietary. | Patients' Payments. | | |
| | | | | | £ | £ | £ | £ | £ | £ |
| 36 | 28 | French ... ... ... ... | 355 | 3,310 | 2,494 | 2,753 | 412 | ... | 3,165 | 400 |
| 20 | 13 | Italian ... ... ... ... | 226 | 2,483 | 696 | 550 | 21 | ... | 571 | ... |
| 51 | 46 | SS. John and Elizabeth ... ... | 91 | ... | 1,531 | 809 | 683 | ... | 1,492 | ... |
| 307 | 259 | The Middlesex ... ... ... | 2,819 | 35,945 | 24,247 | 12,434 | 5,688 | ... | 18,122 | 11,948 |
| 80 | 80 | Alexandra, for Children ... ... | 156 | 104 | 2,896 | 2,120 | 115 | 619 | 2,854 | ... |
| 30 | 29 | Home for Incurable Children ... | 40 | ... | 1,261 | 820 | 15 | 438 | 1,273 | ... |
| 181 | 127 | Hospital for Sick Children ... | 1,414 | 17,155 | 11,148 | 10,565 | 895 | ... | 11,460 | 1,290 |
| 18 | 9 | British Lying-in ... ... ... | 173 | 614 | 1,507 | 622 | 990 | 8 | 1,620 | 180 |
| 56 | 32 | Queen Charlotte's Lying-in ... | 865 | 1,151 | 3,288 | 2,468 | 308 | 22 | 2,790 | 600 |
| 26 | 21 | New Hospital for Women... ... | 253 | 3,847 | 2,108 | 1,513 | 173 | 520 | 2,206 | ... |
| 52 | 48 | Samaritan Free ... ... ... | 504 | 4,489 | 4,882 | 4,442 | 27 | ... | 4,469 | 1,350 |
| 175 | 132 | National, for the Paralysed, etc... | 693 | 3,523 | 10,194 | 5,829 | 1,629 | 1,642 | 9,100 | 1,700 |
| 10 | 10 | West End, for Paralysed, etc. ... | 55 | 724 | 1,347 | 973 | ... | 447 | 1,420 | ... |
| 13 | 6 | Central London Ophthalmic ... | 185 | 8,285 | 1,308 | 561 | 62 | 211 | 834 | ... |
| 20 | 3 | Western Ophthalmic ... ... | 49 | 1,994 | 583 | 359 | 122 | 160 | 641 | ... |
| 36 | 32 | National Orthopædic ... ... | 141 | 714 | 1,191 | 452 | ... | 568 | 1,020 | ... |
| 94 | 52 | London Homœopathic ... ... | 712 | 8,882 | 4,977 | 1,981 | 372 | 2,889 | 5,242 | 643 |
| 20 | 10 | Establishment for Gentlewomen... | 135 | ... | 2,337 | 1,270 | 117 | 943 | 2,330 | 400 |
| 1,225 | 937 | | 11,866 | 93,220 | 77,995 | 50,521 | 11,629 | 8,467 | 70,617 | 18,511 |
| | | Dispensaries. | | | | | | | | |
| ... | ... | Margaret-street Infirmary ... | ... | 2,013 | 445 | 290 | 164 | ... | 454 | ... |
| ... | ... | Portland Town ... ... ... | ... | 1,491 | 162 | 143 | ... | 10 | 153 | ... |
| ... | ... | Portobello-road ... ... ... | ... | 337 | 78 | 42 | ... | 53 | 95 | ... |
| ... | ... | St. John's Wood Provident ... | ... | 2,894 | 478 | 228 | 24 | 270 | 522 | ... |
| ... | ... | St. Marylebone General ... ... | ... | 4,244 | 773 | 390 | 131 | 334 | 855 | ... |
| ... | ... | Western General ... ... ... | .. | 18,746 | 1,521 | 1,111 | 50 | .... | 1,161 | 150 |
| 1,225 | 937 | | 11,866 | 122,945 | 81,452 | 52,725 | 11,998 | 9,134 | 73,857 | 18,661 |

### KENSINGTON AND WEST DISTRICT.

Comprising Kensington, Paddington, Bayswater, Kilburn, Chelsea, Brompton, Fulham, Hammersmith, Chiswick, Brentford, Acton, Ealing, etc.

**Special Note.**—The 16 Hospitals situated in this district contain 1,666, of which 1,340 only were constantly occupied throughout 1888. The total expenditure amounted to £125,502, and the total income to £101,927, leaving a deficiency of £23,575. This would have been increased, of course, very largely, had all the available beds been occupied throughout the year.

| No. of Beds. | No. of Beds Daily Occupied. | Hospitals. | In-patients. | Out-patients. | Total Expenditure. | Income. | | | Total Income. | Legacies over £100 not included. |
|---|---|---|---|---|---|---|---|---|---|---|
| | | | | | | Charitable. | Proprietary. | Patients' Payments. | | |
| | | | | | £ | £ | £ | £ | £ | £ |
| 351 | 319 | St. George's ... ... ... ... | 4,084 | 24,134 | 28,643 | 10,713 | 9,431 | ... | 20,144 | 102,251 |
| 281 | 250 | St. Mary's ... ... ... ... | 3,293 | 27,079 | 20,620 | 11,424 | 2,175 | ... | 13,599 | 7,222 |
| 101 | 85 | West London ... ... ... | 1,307 | 18,514 | 5,430 | 5,251 | 73 | ... | 5,324 | ... |
| 321 | 257 | Hospital for Consumption... ... | 1,631 | 13,337 | 29,966 | 16,814 | 7,899 | ... | 24,713 | 21,317 |
| 23 | 15 | Belgrave, for Children ... .. | 153 | 2,420 | 1,477 | 1,429 | 25 | 5 | 1,459 | 250 |
| 24 | 24 | Cheyne, for Incurable Children ... | 37 | ... | 1,396 | 1,617 | 187 | 216 | 2,020 | ... |
| 27 | 24 | Paddington Green, for Children... | 400 | 10,393 | 4,292 | 3,270 | 206 | 133 | 3,609 | 450 |
| 84 | 69 | Victoria, for Children ... . | 877 | 12,424 | 5,417 | 3,887 | 788 | 339 | 5,014 | 1,307 |
| 63 | 27 | Chelsea, for Women ... ... | 306 | 3,295 | 3,180 | 2,606 | 60 | 694 | 3,360 | 305 |
| 66 | 42 | Hospital for Women ... ... | 496 | 4,510 | 6,615 | 3,763 | 153 | 1,240 | 5,156 | 2,350 |
| 105 | 84 | Cancer ... ... ... ... | 641 | 866 | 8,937 | 7,021 | 1,273 | ... | 8,294 | 12,100 |
| 25 | 21 | National, for Diseases of Heart, etc. | 102 | 2,077 | 2,050 | 1,795 | 23 | 386 | 2,204 | ... |
| 135 | 76 | Female Lock ... ... ... | 642 | ... | 3,741 | 2,843 | ... | 630 | 3,473 | 1,175 |
| 20 | 13 | Male Lock ... ... ... ... | 214 | 9,916 | 1,329 | 252 | ... | 793 | 1,045 | ... |
| 9 | 4 | Royal Ear Hospital ... ... | 94 | 2,760 | 682 | 188 | ... | 550 | 738 | ... |
| ... | ... | National Dental ... ... ... | ... | 25,498 | 898 | 388 | ... | 522 | 910 | ... |
| 31 | 30 | St. Mary Magdalene's Home ... | 62 | 22 | 829 | 389 | 11 | 465 | 865 | ... |
| 1,666 | 1,340 | | 14,339 | 157,285 | 125,502 | 73,650 | 22,304 | 5,973 | 101,927 | 148,727 |
| | | **Dispensaries.** | | | | | | | | |
| ... | ... | Brompton Provident ... ... | ... | 912 | 414 | 163 | 10 | 258 | 431 | ... |
| ... | ... | Chelsea, etc.... ... ... ... | ... | 4,407 | 628 | 435 | 142 | ... | 577 | 1,500 |
| ... | ... | Kensington ... ... ... ... | ... | 5,059 | 749 | 826 | 50 | ... | 876 | ... |
| ... | ... | Kilburn, Maida Vale ... ... | ... | 2,056 | 476 | 515 | 43 | ... | 558 | ... |
| ... | .. | Kilburn Provident ... ... ... | ... | 3,711 | 1,038 | 93 | 77 | 868 | 1,038 | ... |
| ... | ... | Notting Hill Provident ... ... | ... | 1,196 | 254 | 145 | 23 | 76 | 244 | ... |
| ... | ... | Paddington Provident ... ... | ... | 7,035 | 591 | 173 | 24 | 386 | 583 | ... |
| ... | ... | Royal Pimlico Provident ... ... | ... | 12,665 | 849 | 292 | 22 | 559 | 873 | ... |
| ... | ... | SS. Paul and Barnabas ... ... | ... | 2,569 | 495 | 363 | ... | 134 | 497 | 200 |
| ... | .. | Westbourne Provident ... ... | ... | 3,595 | 354 | 183 | 7 | 285 | 475 | ... |
| 1,666 | 1,340 | | 14,339 | 200,490 | 131,350 | 76,838 | 22,702 | 8,539 | 108,079 | 150,427 |

### ISLINGTON AND NORTH-WEST DISTRICT.

Comprising Islington, Holloway, Highbury, Hampstead, Highgate, St. Pancras, Stoke Newington, Tottenham, etc.

**Special Note.**—Though one of the largest and most populous districts of the metropolis, it only contains 822 beds (58 more than last year), of which 550 were constantly occupied. The total income amounted to £52,537, and the total expenditure to £58,238, leaving a deficiency of £5,701, which would have been very largely increased had all the beds been occupied.

| No. of Beds. | No. of Beds Daily Occupied. | Hospitals. | In-patients. | Out-patients. | Total Expenditure. | Income. | | | Total. Income. | Legacies over £100 not included. |
|---|---|---|---|---|---|---|---|---|---|---|
| | | | | | | Charitable. | Proprietary. | Patients' Payments. | | |
| | | | | | £ | £ | £ | £ | £ | £ |
| 68 | 48 | Great Northern Hospital ... ... | 654 | 12,726 | 4,795 | 3,615 | 372 | ... | 3,987 | 4,150 |
| 47 | 43 | North-West London ... ... | 650 | 13,788 | 4,020 | 5,409 | 75 | 101 | 5,585 | ... |
| 103 | 62 | Tottenham ... ... ... ... | 721 | 7,836 | 4,214 | 2,986 | 518 | 594 | 4,098 | ... |
| 207 | 169 | University College ... ... ... | 2,701 | 35,786 | 19,900 | 9,071 | 5,222 | 33 | 14,326 | 4,312 |
| 40 | 37 | North London Consumption ... | 316 | 3,037 | 4,511 | 4,353 | 80 | 25 | 4,458 | 450 |
| 220 | 83 | London Fever ... ... ... | 742 | ... | 10,180 | 6,327 | 1,597 | 2,430 | 10,354 | 2,140 |
| 25 | 23 | Hospital for Epilepsy, etc. ... | 89 | 757 | 2,533 | 1,222 | ... | 817 | 2,039 | 1,150 |
| 69 | 58 | London Temperance ... ... | 742 | 2,538 | 5,249 | 4,728 | 353 | 226 | 5,307 | 758 |
| 15 | 9 | Hampstead Home Hospital ... | 107 | 25 | 1,854 | 675 | 463 | 439 | 1,577 | ... |
| 28 | 18 | Invalid Asylum ... ... ... | 185 | ... | 982 | 539 | 70 | 197 | 806 | 500 |
| 822 | 550 | | 6,907 | 76,493 | 58,238 | 38,925 | 8,750 | 4,862 | 52,537 | 13,460 |
| | | **Dispensaries.** | | | | | | | | |
| ... | ... | Child's Hill ... ... ... ... | ... | 633 | 227 | 41 | 11 | 181 | 233 | ... |
| ... | ... | Hampstead Provident ... ... | ... | 9,993 | 932 | 352 | 11 | 576 | 939 | ... |
| ... | ... | Holloway and North Islington ... | ... | 5,263 | 921 | 504 | ... | 434 | 938 | 110 |
| ... | ... | Islington ... ... ... ... | ... | 14,314 | 860 | 288 | 6 | 527 | 821 | ... |
| ... | ... | St. Pancras and Northern ... | ... | 2,962 | 593 | 345 | 121 | 80 | 546 | 300 |
| ... | ... | Stamford Hill ... ... ... | ... | 7,171 | 593 | 489 | 104 | ... | 593 | 380 |
| 822 | 550 | | 6,907 | 116,829 | 62,364 | 40,944 | 9,003 | 6,660 | 56,607 | 14,250 |

### STRATFORD AND EAST-END DISTRICT.

Comprising Bethnal Green, Tower Hamlets, West Ham, Whitechapel, Hackney, Stepney, Limehouse, Poplar, and the East.

**Special Note.**—This district contains 1,309 beds, of which 1,036 were in constant occupation. The income was slightly in excess of the expenditure this year, though in 1887 the managers had to face the heavy deficit of £15,400.

| No. of Beds. | No. of Beds Daily Occupied. | Hospitals. | In-patients. | Out-patients. | Total Expenditure. | Income. | | | Total Income. | Legacies over £100 not included. |
|---|---|---|---|---|---|---|---|---|---|---|
| | | | | | | Charitable. | Proprietary. | Patients' Payments. | | |
| | | | | | £ | £ | £ | £ | £ | £ |
| 125 | 112 | German ... ... ... ... | 1,284 | 19,891 | 9,246 | 6,892 | 2,275 | 247 | 9,414 | 2,970 |
| 776 | 640 | London ... ... ... ... | 8,268 | 101,548 | 55,078 | 38,158 | 21,418 | 55 | 59,631 | 10,504 |
| 50 | 35 | Poplar ... ... ... ... | 651 | 10,078 | 3,002 | 2,942 | 380 | ... | 3,322 | 420 |
| 164 | 109 | City of L'nd'n for Diseases of the Chest | 972 | 14,856 | 10,199 | 6,295 | 475 | ... | 6,770 | 4,582 |
| 102 | 75 | East London for Children... ... | 1,003 | 18,265 | 6,909 | 8,422 | 422 | ... | 8,844 | 785 |
| 92 | 65 | Mrs. Gladstone's Home ... ... | 1,078 | ... | 1,450 | 933 | 383 | ... | 1,316 | 500 |
| 1,309 | 1,036 | | 13,256 | 164,638 | 85,884 | 63,642 | 25,353 | 302 | 89,297 | 19,761 |
| | | Dispensaries. | | | | | | | | |
| ... | ... | Eastern ... ... ... ... | ... | 5,075 | 574 | 272 | 242 | 46 | 560 | ... |
| ... | .. | London ... ... ... ... | ... | 3,002 | 454 | 152 | 176 | ... | 328 | ... |
| ... | ... | Queen Adelaide's ... ... ... | ... | 4,295 | 468 | 562 | 145 | ... | 707 | ... |
| ... | ... | Tower Hamlets ... ... ... | ... | 3,762 | 507 | 306 | 34 | 130 | 470 | ... |
| ... | ... | West Ham, etc. ... ... ... | ... | 18,038 | 860 | 878 | 158 | ... | 1,036 | ... |
| 1,309 | 1,036 | | 13,256 | 198,810 | 88,747 | 65,812 | 26,108 | 478 | 92,398 | 19,761 |

### SUMMARY.

It will be seen from the following summary that the voluntary Hospitals and Medical Charities of London, during the 12 months ending 31st December, 1888, relieved over one million one hundred thousand patients, at a cost of £562,681. The ordinary income only amounted to £508,826, and leaving a deficiency of £53,855 on the year's work for the suffering poor of London.

| No. of Beds. | No. of Beds Daily Occupied. | Hospitals and Dispensaries. | In-patients. | Out-patients. | Total Expenditure. | Income. | | | Total Income. | Legacies not included. |
|---|---|---|---|---|---|---|---|---|---|---|
| | | | | | | Charitable. | Proprietary. | Patients' Payments. | | |
| | | | | | £ | £ | £ | £ | £ | £ |
| 1,461 | 948 | Newington and South District | 13,123 | 128,430 | 81,567 | 31,355 | 37,670 | 9,031 | 78,056 | 4,670 |
| 663 | 413 | City and West Central District | 6,597 | 139,564 | 47,312 | 28,260 | 8,531 | 4,175 | 40,966 | 10,682 |
| 866 | 702 | Westminster District ... ... | 8,228 | 131,359 | 69,889 | 39,999 | 11,292 | 7,572 | 58,863 | 12,933 |
| 1,225 | 937 | St. Marylebone and West Central District ... ... ... | 11,866 | 122,945 | 81,452 | 52,725 | 11,998 | 9,134 | 73,857 | 18,661 |
| 1,666 | 1,340 | Kensington and West District... | 14,339 | 200,490 | 131,350 | 76,838 | 22,702 | 8,539 | 108,079 | 150,427 |
| 822 | 550 | Islington and North-West District | 6,907 | 116,829 | 62,364 | 40,944 | 9,003 | 6,660 | 56,607 | 14,250 |
| 1,309 | 1,036 | Stratford and East End District | 13,256 | 198,810 | 88,747 | 65,812 | 26,108 | 478 | 92,398 | 19,761 |
| 8,012 | 5,926 | | 74,316 | 1,038,427 | 562,681 | 335,933 | 127,304 | 45,589 | 508,826 | 231,384 |

### Amusements and Relaxation.

### Word Competition.

Commenced April 6th, Ends June 29th.

Three Prizes of 15s., 10s., 5s., will be given for the largest number of words derived from the words set for dissection.

Proper names, abbreviations, foreign words, words of less than four letters, and repetitions are barred; plurals, and past and present participles of verbs, are allowed. Nuttall's *Standard* dictionary only to be used.

N.B.—Word dissections must be sent in WEEKLY, arranged alphabetically, with correct total affixed.

The word for dissection for this, the *Eleventh* week of the quarter, being

"PORTLAND."

### Notice to all Correspondents.

N.B.—All letters referring to this page which do not arrive at 139 and 140, Salisbury-court, London, E.C., by the *first post on Thursdays*, and are not addressed PRIZE EDITOR, will in future be disqualified and disregarded.

Competitors can enter for *all* quarterly competitions, but no competitor may take more than *two quarterly prizes* during the year.

### Results of Ninth Week.

| Names. | June 6th. | Totals. | Names. | June 6th. | Totals. |
|---|---|---|---|---|---|
| Patience | 11 | 635 | N'importe Qui | 9 | 506 |
| Nurse Duty | 12 | 618 | Pollie | 11 | 472 |
| Lightowlers | 11 | 617 | W. G. R. | 11 | 467 |
| Scrooge | — | 541 | Robe | — | 150 |
| Reldas | — | 250 | Beryl | — | 391 |
| Tinie | 11 | 635 | Nesta | — | 124 |
| K. G. | 11 | 589 | Daffodil | — | 89 |
| Broxbourne | 12 | 607 | A Schoolgirl | 10 | 202 |
| Lauriston | 11 | 609 | Montague | — | 79 |
| Spes | 10 | 589 | Sheeny | — | 111 |
| Gretta | — | 224 | Alice R. | — | 63 |
| Francesca | — | 476 | Belfast | — | 163 |
| Heatherbell | — | 185 | Gentian | — | 209 |
| Sycamore | — | 201 | Hope | — | 39 |
| Arctus | — | 499 | Louie | — | 139 |
| Amicus | 12 | 592 | Hamilton | — | 121 |
| Scratcher | — | 197 | E. M. | — | 120 |
| Jemima | — | 138 | F. J. D. | — | 112 |
| Concours | — | 427 | Ladyr | — | 102 |
| C. J. S. | — | 134 | Surgical Nurse | — | 128 |
| See-Saw | 11 | 550 | L. Pearce | — | 32 |
| Rosemont | — | 173 | Contentio | — | 98 |
| Jenny Wren | 12 | 372 | M. W. | 11 | 57 |
| Eric | 9 | 496 | Esperance | 11 | 58 |
| Tiny | 12 | 544 | H. B. | 10 | 10 |

